# Serine Phosphorylation of SLP76 Is Dispensable for T Cell Development but Modulates Helper T Cell Function

**DOI:** 10.1371/journal.pone.0170396

**Published:** 2017-01-20

**Authors:** Victor H. Navas, Céline Cuche, Andres Alcover, Vincenzo Di Bartolo

**Affiliations:** 1 Lymphocyte Cell Biology Unit, Institut Pasteur, Paris, France; 2 CNRS URA 1961, Paris, France; 3 Université "Pierre et Marie Curie", Paris, France; 4 INSERM U1221, Paris, France; Universite Paris-Sud, FRANCE

## Abstract

The adapter protein SLP76 is a key orchestrator of T cell receptor (TCR) signal transduction. We previously identified a negative feedback loop that modulates T cell activation, involving phosphorylation of Ser376 of SLP76 by the hematopoietic progenitor kinase 1 (HPK1). However, the physiological relevance of this regulatory mechanism was still unknown. To address this question, we generated a SLP76-S376A-expressing knock-in mouse strain and investigated the effects of Ser376 mutation on T cell development and function. We report here that SLP76-S376A-expressing mice exhibit normal thymocyte development and no detectable phenotypic alterations in mature T cell subsets or other lymphoid and myeloid cell lineages. Biochemical analyses revealed that mutant T cells were hypersensitive to TCR stimulation. Indeed, phosphorylation of several signaling proteins, including SLP76 itself, phospholipase Cγ1 and the protein kinases AKT and ERK1/2, was increased. These modifications correlated with increased Th1-type and decreased Th2-type cytokine production by SLP76-S376A T cells, but did not result in significant changes of proliferative capacity nor activation-induced cell death susceptibility. Hence, our results reveal that SLP76-Ser376 phosphorylation does not mediate all HPK1-dependent regulatory effects in T cells but it fine-tunes helper T cell responses.

## Introduction

Adaptive immune responses are initiated upon recognition by the T cell receptor (TCR) of peptide antigen–major histocompatibility complex (MHC) complexes, displayed on the surface of antigen-presenting cells. TCR engagement results in a rapid activation of protein tyrosine kinases e.g. Lck and ZAP-70 [1] that, in turn, phosphorylate two key scaffold proteins, LAT [2] and SLP76 [3]. Phosphorylated LAT recruits SLP76 via the GRB2-related adaptor GADS [4], thus nucleating a central hub that gathers a wide array of effectors to activate downstream signaling pathways. Hence, assembly and stability of this proximal signaling platform critically affect the outcome of immune responses. For instance, both LAT and SLP76 have been implicated in the control of T cell cytoskeleton reorganization, generation of second messengers and activation of transcription factors [5–9], thus driving T cell proliferation, differentiation and specific effector functions.

As mentioned above, protein-protein interactions dependent on tyrosine phosphorylation play a central role in the assembly of signaling complexes. Conversely, several mechanisms have been described that contribute to their dissociation, leading to downmodulation or termination of T cell activation. These include recruitment of tyrosine phosphatases [10], ubiquitylation [11, 12] or serine/threonine inhibitory phosphorylation [13] of critical components of the TCR signaling machinery. Of note, manipulation of similar regulatory mechanisms has a potential interest in improving the efficacy of adoptive immunotherapy, e.g. against cancer [14, 15]

We previously identified a negative feedback loop downregulating TCR signaling and T cell activation, that involves Ser/Thr phosphorylation of SLP76 and GADS by the Hematopoietic Progenitor Kinase (HPK)1 [13, 16], a member of the Germinal Center Kinase family. We have shown that when HPK1 is recruited in early signaling complexes, through its binding to the SLP76 SH2 domain [17], it induces the phosphorylation of Ser376 in SLP76 and Thr262 in GADS. These post-translational modifications prompt the interaction of a 14-3-3 protein dimer with the SLP76-GADS complex and consequently lead to its dissociation from LAT. We have also shown that this mechanism negatively regulates tyrosine phosphorylation of phospholipase Cγ1 (PLCγ1) and SLP76, as well as NFAT-dependent transcriptional activity in TCR-stimulated T cell lines [13]. However, the physiological relevance *in vivo* of this regulatory feedback loop was not investigated. To address this question, we have generated a knock-in mouse strain expressing a SLP76-S376A mutant in place of wild-type SLP76 and used this model to investigate whether T cell development and T cell responses are modified by impairing this negative regulatory mechanism. Immunophenotypic analyses did not reveal significant alterations in thymocyte development or homeostasis of T cells in SLP76-S376A mice. Other lymphoid or myeloid cell lineages also appeared unaffected in this strain. However, *in vitro* stimulated SLP76-S376A T cells were hyper-responsive as compared to wild-type T cells, since phosphorylation of several signaling proteins, including SLP76, PLCγ1 and the kinases AKT, ERK1 and ERK2 was increased in the former. Conversely, JNK and p38 pathways of mitogen-activated protein (MAP) kinases were not affected in mutant T cells. *In vitro* functional assays revealed some differences in Th1 and Th2 cytokine production by activated SLP76-S376A T cells, whereas induction of proliferation or activation-induced cell death were not modified.

Collectively, our analyses of SLP76-S376A knockin mice indicate that HPK1-induced 14-3-3 binding to SLP76 leads to qualitative and quantitative changes in TCR signaling that affect cytokine production, while other functional responses of CD4^+^ T cells remain unaltered.

## Materials and Methods

### Mice

All mice were housed in the Central Animal Facility of the Institut Pasteur, under specific pathogen-free conditions. C57Bl/6J mice were purchased from Janvier Labs (Le Genest St. Isle, France). The SLP76-S376A knockin strain was generated by the CIPHE facility (Centre d'Immunologie de Luminy, Marseille, France). Briefly, a sequence encompassing exons 15 to 20 of the murine *Lcp2* gene was isolated from a C57Bl/6 BAC library and subcloned in a pBluescriptII KS+ vector. A TCC codon (corresponding to Ser 376) was mutated to GCC (Ala) by PCR in exon 17. Two additional silent point mutations were added in order to generate an *AfeI* restriction site, allowing the detection of the mutant allele after PCR amplification (see S1 Fig). Mutant allele was subcloned in a targeting vector containing a *lox*P-tACE-CRE-PKG-gb2-*neo*^r^ cassette, enclosed by *lox*P sites. This construct was electroporated in CK35 ES cells (129/sv strain) and positive clones were screened by southern blot. Selected clones were injected in FVB blastocysts that were subsequently implanted. Chimeric animals were screened and selected for germinal transmission. The murine angiotensin-converting enzyme (tACE) promoter drives transcription of the CRE recombinase in the male germline, thus inducing the excision of the selection cassette in this tissue. Mice were then backcrossed on the C57Bl/6j strain for at least 10 generations before experiments. Mice between 7 and 10 weeks of age were used in compliance with the European Communities recommendations. This study was approved by the Ethical & Animal Experimentation Committee of the Institut Pasteur (CETEA 89) and the French Ministry of Higher Education and Research (project nr. 00311.02).

### Antibodies

Antibodies and reagents used for flow cytometry were: CD8-APC, Fixable viability stain 450, antiCD16/32 (eBiosciences); anti-rabbit-PE (Jackson Immunoresearch); anti-SLP76 (pY128), CD4-PE, CD25-FITC, CD8 FITC and anti-mouse IgG1-PE (BD Biosciences); CD4-PEVio770 (Miltenyi Biotec); CD3-BV421 (Biolegend); anti-pErk1/2 (pT202/pY204) (Cell signaling Technology). The following reagents were used for cell stimulation, immunoblotting or immunoprecipitation: anti-CD3-biontinylated, anti-CD28-biontinylated, anti-CD3ε, anti-CD28 (eBiosciences); streptavidin (Sigma); anti-pErk1/2 (pT202/pY204), anti-pPLCγ1 (pY783), anti-pp38 (pT180/pY182), anti-pJNK (pT183/Y185), anti-pAkt (pS473), anti-pSLP76 (pS376) (Cell Signaling Technology); anti-GADS (Santa Cruz Biotechnology Inc.); rabbit anti-SLP76 or goat-anti-SLP76 (Thermo Fisher Scientific); anti-β-tubulin (Chemicon); for anti-14-3-3 immunoblotting we used a mix of anti-pan 14-3-3 and anti-14-3-3ζ (Santa Cruz, Biotechnology Inc.).

### T cell isolation and purification

T cells were isolated from pooled lymph nodes (inguinal, axillary, submandibular and mesenteric). Organs were dissociated and T cells were purified using a CD4^+^ T cell isolation kit (Miltenyi Biotec) and AutoMACS Pro Separator for automated negative cell sorting.

### T cell proliferation and apoptosis assays

3x10^5^ purified CD4^+^ T cells were stimulated in a 96-well plate pre-coated with anti-CD3 antibodies (1 μg/ml) and soluble anti-CD28 antibodies (1 μg/ml) for 72 h incubated at +37°C. In indicated experiments purified T cells were labeled with 5 μM CFSE (Invitrogen) previous to stimulation, for assessment of cell proliferation. 7AAD-AnnexinV tandem staining was performed after 72 h of *in vitro* stimulation, according to the manufacturer’s instructions (BD Pharmingen).

### Immunoblotting and immunoprecipitation

CD4+ T cells were purified from lymph nodes and incubated for 30 min on ice with 10 μg/ml of biotin-conjugated anti-CD3ε and anti-CD28. Cells were washed in cold medium, resuspended in pre-warmed medium containing streptavidin (10 μg/ml) and incubated at 37°C for the indicated time points. Activation was stopped by transferring cells into ice-cold PBS containing sodium-orthovanadate (2 mM) and sodium azide (0.02%). Cell pellets were lysed in buffer containing 0.25% lauryl-β-maltoside, 50 mM Tris, 140 mM NaCl, 1 mM EGTA and a cocktail of protease and phosphate inhibitors. Insoluble material was removed by centrifugation at 20,800xg for 10 min at 4°C. Samples were either stored at –20°C or directly fractionated by electrophoresis on Novex NuPage gels (Life Technologies) using MOPS buffer, then transferred to nitrocellulose membranes and analyzed by immunoblotting, as described [13, 16].

For immunoprecipitation, goat anti-SLP76 antibodies (Thermo Scientific) were covalently coupled to Protein-A/G Plus agarose beads using the Pierce Classic IP kit (Thermo Scientific), according to the manufacturer instructions. Total lymph node cells or purified CD4^+^ T cells were activated with crosslinked anti-CD3 antibodies for various time points as described above, then lysed with RIPA buffer (Cell Signaling Technology) supplemented with a phosphatase and protease inhibitor cocktail. After centrifugation at ≥13000xg for 10 min at 4°C to remove nuclei and insoluble material, lysates were incubated overnight with anti-SLP76-coated beads at +4°C under continuous rotation. Beads were then washed thrice with RIPA buffer, then supernatant was removed and beads incubated in reducing NuPage gel loading buffer (Life Technologies) for 10 min at +70°C. Supernatants were either stored at –20°C or directly analyzed by gel electrophoresis and immunoblotting as outlined above.

### *In vitro* T cell differentiation and detection of cytokine secretion

CD4^+^ T cells were isolated from lymph nodes by negative selection using a CD4^+^ T cell isolation kit (Miltenyi Biotec). Purified CD4^+^ T cells were then stained with CD44-PE/Cy7 and CD62L-APC/Cy7, or CD44-APC and CD62L-PE (eBiosciences). The CD44^low^CD62L^high^ naive CD4^+^ T cell population was purified with an Aria III cell sorter (BD Bioscience). 10^5^ naive T cells/well were stimulated in 96-well plates coated with anti-CD3 (1 μg/ml) (eBiosciences) and soluble anti-CD28 (1 μg/ml) (eBiosciences). IL-2 (20 ng/ml), IL-12 (20 ng/ml) and blocking anti-IL4 antibodies (10 μg/ml; Biolegend) were added to cell culture for differentiation into Th1 cells. IL-2 (20 ng/ml), IL-4 (100 ng/ml) and blocking antibodies against IFNγ (10 μg/ml; Biolegend) and IL12 (10 μg/ml; Biolegend) were added for differentiation into Th2. After 5-day culture at +37°C, differentiated T cells were washed with PBS and restimulated in plates coated with anti-CD3 (1 μg/ml) in the presence of soluble anti-CD28 (1 μg/ml) and IL-2 (20 ng/ml). Supernatants were harvested 24 hours after restimulation and secreted IL-4 and IFNγ levels were measured by specific ELISA kits (R&D Systems). Assays were performed according to manufacturer’s instructions.

### Flow cytometery

Flow cytometry experiments were performed using either a LSR Fortessa (Beckton Dickinson) or MACSQuant Analyzer (Miltenyi biotech). Flow cytometry data were analyzed using FlowJo software (FlowJo, LLC). Cells were isolated and incubated with anti-CD16/32 to block Fc receptors, then incubated with the appropriate combination of fluorescent-labeled antibodies. For intracellular staining, cells were stimulated by CD3/CD28 crosslinking as explained above and activation was stopped by adding paraformaldehyde at 4% final concentration. After 15 min at RT, cells were incubated with anti-CD16/32, surface stained with anti-CD4, then permeabilized with methanol, overnight at -20°C. After washing, cells were incubated with anti-SLP76 pY128 and anti-pERK1/2, followed by anti-mouse-IgG1-PE and anti-rabbit IgG-AlexaFluor647.

Multiparametric immunophenotyping was performed at the CIPHE-PHENOMIN (Inserm, US012) flow cytometry facility. Leukocytes from spleen and thymus were extracted as described on IMPRESS protocol (https://www.mousephenotype.org/impress/protocol/174/7). Briefly, organs were disrupted on OctoGentleMACS system (Miltenyi Biotec) with 600 Mendel Unit Collagenase D (Roche Life Science) and 30 μg DNAse I (Sigma) for 20 min at RT. Cell suspension was filtered and counted. Red blood cells were lysed for 1 min at RT using Ammonium-Chloride-Potassium (ACK) lysis solution (eBioscience). Before staining, cells were pre-incubated 10 min on ice with the 2.4G2 antibody to block Fc receptors. In all experiment, Sytox Blue (Invitrogen) was used to exclude dead cells from the analysis. Multiparameter FACS acquisition was performed on a Fortessa LSRII SORP system (BD Biosciences). Analysis was performed using FACSDiva 8.01 (BD Biosciences) software. Doublets were systematically excluded based on side scatter (SSC) and forward scatter (FSC) parameters. Antibodies used for immunophenotyping are listed in S1 Table.

### Statistics

Two-tailed t-test, Mann-Whitney test or two-way ANOVA were used to assess the statistical significance of our results. These tests were performed using the GraphPad Prism 5.0 software.

## Results

### Generation of SLP76-S376 knock-in mice

In order to address the physiological relevance of the negative feedback loop involving HPK1, SLP76 and 14-3-3 proteins, we generated an SLP76-S376A knock-in mouse strain using ES targeted mutagenesis (S1 Fig, panels A, B). Mice were generated on a mixed 129/sv x C57Bl/6 background then backcrossed on the C57Bl/6 strain. Mutant mice were born at expected mendelian frequency, they were healthy and fertile and had a normal lifespan. Immunoblot analysis with phospho-Ser376-specific antibodies confirmed that phosphorylation of SLP76 at this site was undetectable in mutant mice (S1 Fig, panel C). Noteworthy, we did not find significant differences between the expression of wild-type and mutant SLP76 (S1 Fig, panel D), suggesting that S376A mutation does not perturb protein stability at steady state.

**Fig 1:**
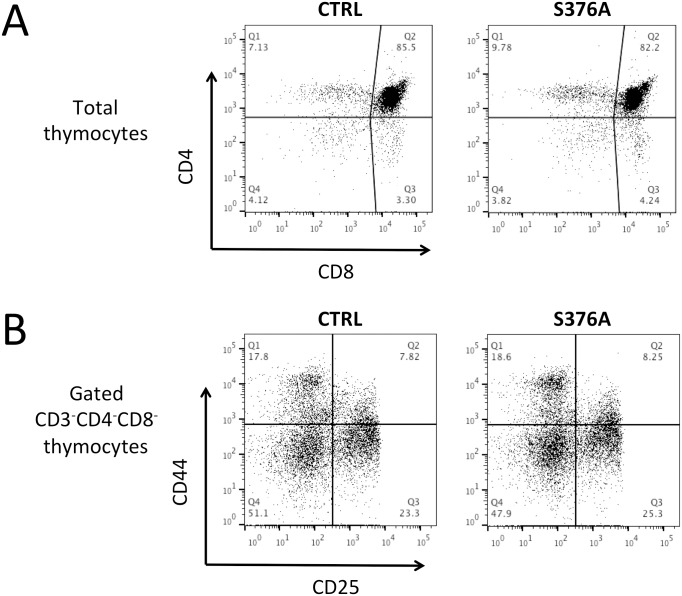
Analysis of thymic development and peripheral lymphoid cell frequencies in control and SLP76-S376A mice. **A.** Thymocytes isolated from wild type or control mice SLP76-S376A were stained with anti-CD3, CD4 and CD8 antibodies and analyzed by flow cytometry. **B.** Thymocytes were stained with antibodies against CD3, CD4, CD8, CD44 and CD25 and analyzed by flow cytometry. Gated CD3^-^CD4^-^CD8^-^ cells were analyzed for expression of CD44 and CD25. One representative experiment out of 3 performed is shown. See Table 1 for average data from all experiments.

Since SLP76 is an essential regulator of T cell lineage development [3], we initially addressed the potential effect of Ser376 mutation on thymocyte maturation. We found that the cellularity and the frequencies of CD4^+^ and CD8^+^ single-positive, CD4^+^CD8^+^ double-positive and CD4^-^CD8^-^ thymocytes in SLP76-S376A mice were comparable to those observed in control mice (Fig 1A and Table 1A). Moreover, immature thymocyte subpopulations, as defined by CD44 and CD25 expression in the triple negative CD3^-^CD4^-^CD8^-^ compartment [18], were not altered in SLP76-S376A mice (Fig 1B and Table 1B). These data suggest that SLP76-S376A expression does not significantly affect T cell development. Nevertheless, a high-content screening by multicolor flow cytometry was performed to further extend this analysis. This screening did not reveal significant alterations in the frequencies of the major subpopulations of thymic αβ or γδ T cells, regulatory T cells or NK cells (S2 Fig; marker definition of each cell population analyzed is given in S2 Table), hence supporting the absence of noticeable effects of SLP76-S376A on T cell generation and maturation.

**Table 1:** Thymocytes subpopulation frequencies in control or SLP76-S376A knock-in mice. Data represent percentage of the indicated subpopulation (average ± SD; n = 8).

**1A**		
	**CTRL** ^§^	**S376A** ^§^
CD4^-^CD8^-^ (DN)	4.13 ± 0.22	4.11 ± 0.66
CD4^+^ CD8^+^ (DP)	83.45 ± 2.02	80.4 ± 2.97
CD4^+^	8.89 ± 1.54	10.99 ± 1.72
CD8^+^	3.71 ± 0.43	4.78 ± 0.75
**1B**		
	**CTRL** ^#^	**S376A** ^#^
CD44^+^CD25^-^ (DN1)	17.55 ± 1.68	20.05 ± 1.31
CD44^+^CD25^+^ (DN2)	10.06 ± 3.03	9.13 ± 2.19
CD44^-^CD25^+^ (DN3)	26.25 ± 3.87	23.3 ± 1.75
CD44^-^CD25^-^ (DN4)	46.55 ± 5.61	45.2 ± 4.19

^§^ % of total thymocytes

^#^ % of gated CD3^-^CD4^-^CD8^-^

**Fig 2:**
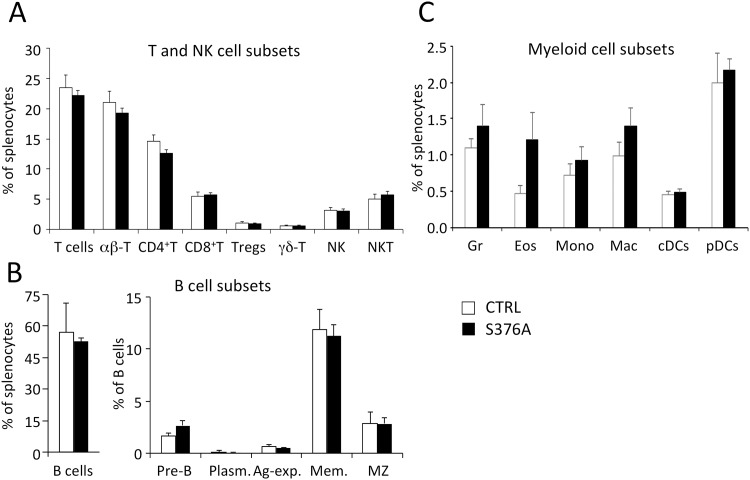
Analysis of hematopoietic lineage cells in the spleen of control and SLP76-S376A mice. Splenocytes isolated from wild type (open bars) or SLP76-S376A (filled bars) were stained for detection of main T and NK cell (**A**), B cell (**B**) or myeloid cell (**C**) sub-populations, then analyzed by flow cytometry. Histograms represent the mean frequencies of each population in total splenocytes (A,C) or within B cells (B, right panel). Error bars represent SD (n = 6). Plasm.: plasma cells, Ag-exp.: antigen-experienced B cells. Mem.: memory B cells; MZ: marginal zone B cells. Gr: granulocytes; Eos: eosinophils; Mono: monocytes; Mac: macrophages; cDCs: conventional dendritic cells; pDCs: plasmacytoid dendritic cells. See S2 Table for marker definition of each cell type.

We then analyzed the peripheral lymphoid compartment of SLP76-S376A mice. We found that the size and cellularity of spleens in mutant mice were comparable to those observed in control C57Bl/6j mice (136.3±16.2x10^6^ cells/spleen for controls vs 151.2±30,4x10^6^ for SLP76-S376A; average±SD with n = 6; p = 0.319). All major populations of T, NK and B cells were present in comparable frequencies in the spleen of both mouse strains (Fig 2A and 2B), suggesting that no major alteration of lymphoid cell lineages was induced by SLP76-S376A expression under homeostatic conditions. We also analyzed the main populations of myeloid cells in the spleen since SLP76 is broadly expressed in cells of hematopoietic origin [19]. Again, most cell lineages did not appear to be altered in SLP76-S376A knock-in mice when compared to control animals (Fig 2C). Although the frequency of eosinophils in the spleen of SLP76-S376A mice appeared to be increased compared to control mice (Fig 2C), further experiments failed to detect a significant difference in eosinophil counts in the blood or peripheral lymphoid organs of control and SLP76-S376A mice (data not shown).

Altogether, these data indicate that mutation of Ser376 in SLP76 has no evident impact on the development and homeostasis of the major lineages of hematopoietic cells.

### T cells from SLP76-S376A knock-in mice show impaired recruitment of 14-3-3 to SLP76-containing signaling complexes

**Fig 3:**
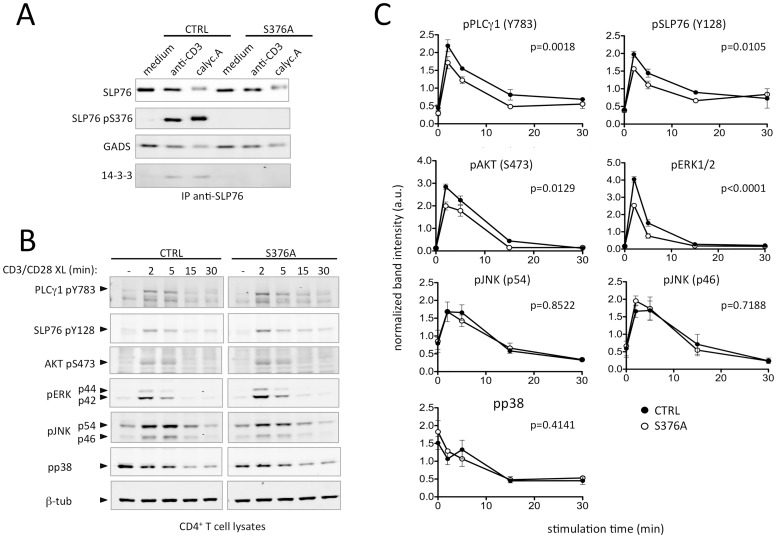
Ser376 mutation impairs 14-3-3 binding to SLP76 and affects activation of several T cell signalling pathways. **A.** Total lymph node cells from wild type (CTRL) or mutant (S376A) mice were isolated and either left unstimulated (medium) stimulated by anti-CD3 antibody crosslinking (CD3) or treated with 50 μM calyculin A (calyc.A) for 10 min at +37°C. Cells were then lysed and soluble protein extracts analysed by immunoblotting with the indicated antibodies. **B.** CD4^+^ T cells purified from lymph nodes of wild type and SLP76-S376A mice were left unstimulated (-) or stimulated by anti-CD3 plus anti-CD28 antibody crosslinking for the indicated time points. Cells were subsequently lysed and analysed by immunoblotting using the indicated antibody. Images were acquired with an Odyssey infrared scanner (LI-COR) and quantified. Data are representative of two independent experiments. **C.** Quantification of signaling protein phosphorylation. For each phosphoprotein, band intensities were quantified as described in B and normalized by the β-tubulin relative amount in the same lane. Normalized intensities were then divided by the mean normalized intensity of the same experiment. Each data point represents mean±SEM from three independent experiments. Statistical analysis was performed by two-way ANOVA. The p-value indicating the significance of the difference between the two curves (CTRL vs S376A) is indicated in each panel. A.u.: arbitrary units.

We previously reported, using Jurkat T cells, that disabling the recruitment of 14-3-3 to SLP76 by mutation of Ser376 reinforced TCR signaling and cell activation [13]. To address whether this Ser376-dependent negative regulatory mechanism is also relevant in primary T lymphocytes, we isolated lymph node cells from control or SLP76-S376A mice. Cells were left unstimulated, treated with anti-CD3 antibodies or the phosphatase inhibitor calyculin A, then they were lysed and immunoprecipitated with anti-SLP76 antibodies. In line with our previous findings, either treatment induced phosphorylation of Ser376 in wild type but not mutant SLP76 (Fig 3A). Accordingly, 14-3-3 proteins co-precipitated with Ser376-phosphorylated SLP76 from control T cells, whereas this interaction was undetectable in SLP76-S376A T cells. On the other hand, GADS was constitutively bound to SLP76 in resting and stimulated cells, and this interaction was not impaired by mutation of Ser376, in agreement with our former work [16]. Comparable results were obtained with purified murine CD4^+^ T cells (see below). Hence, this finding indicates that mutation of Ser376 is sufficient to prevent 14-3-3 recruitment to the SLP76-GADS complex in mouse T cells.

### SLP76-S376A T cells show increased TCR-induced activation of selected signaling pathways

Based on our previous results [13, 16], we expected that impairment of SLP76 interaction with 14-3-3 would increase the activation of downstream signaling effectors. Thus, we looked for potential changes in the phosphorylation of key elements of TCR-induced signaling pathways.

**Fig 4:**
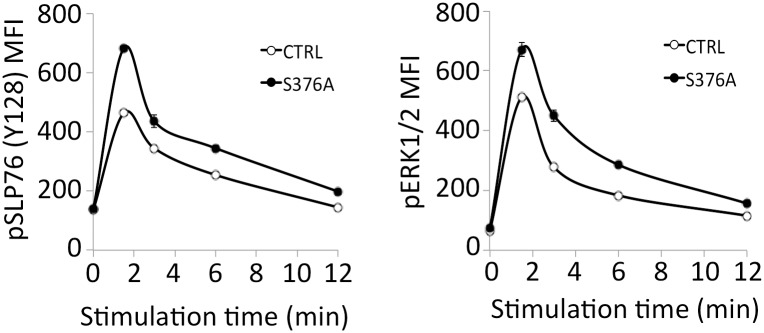
Intracellular flow cytometry analysis of SLP76 and ERK1/2 phosphorylation in control and SLP76-S376A T cells. Lymph node cells stimulated by anti-CD3 crosslinking were fixed and permeabilized, then stained with anti-CD4 and anti-CD8 antibodies, and antibodies against phosphorylated SLP76 (Y128; upper panel) or ERK1/2 (lower panel). Plots show mean fluorescent intensities for CD4^+^ cells within the lymphocyte gate. Data are representative of three independent experiments.

To this aim, CD4^+^ T cells were purified from lymph nodes of wild type and SLP76-S376A mice, then activated *in vitro* by anti-CD3 and CD28 antibody crosslinking for several time points. Cells were subsequently lysed and protein extracts analyzed by gel electrophoresis and immunoblotting with phosphospecific antibodies. Band intensities were quantified and normalized to β-tubulin amount in each lane. As shown in Fig 3B, stimulation-induced phosphorylation of Tyr128 of SLP76 was higher in SLP76-S376A T cells compared to control cells. Similarly, phosphorylation of PLCγ1 at Tyr783 was increased in mutant CD4^+^ T cells, particularly at early time points of stimulation (Fig 3B and 3C). Of note, increased tyrosine phosphorylation of both SLP76 and PLCγ1 dependent on Ser376 mutation is consistent with our previous data in human T cells [13, 16]. Moreover, increased AKT phosphorylation at Ser473 as well as ERK1 and ERK2 kinases phosphorylation was observed in mutant T cells. The latter result can be a direct consequence of the increased activation of PLCγ1, which acts as an upstream activator of the RAS-RAF-MEK-ERK1/2 signaling cascade through the production of diacylglycerol [20]. SLP76 and ERK phosphorylation was also analyzed in anti-CD3-stimulated cells by intracellular staining and flow cytometry. These experiments confirmed the increased phosphorylation of both proteins in SLP76-S376A-expressing CD4^+^ T cells (Fig 4).

Surprisingly, inducible phosphorylation of p38 and JNK MAP kinases was not significantly different in SLP76-S376A relative to control T cells (Fig 3B and 3C), thus indicating that expression of SLP76-S376A results in qualitative differences rather than a general increase of T cell activation.

### Proliferation and activation-induced cell death are not affected in SLP76-S376A T cells

To address whether the signaling modifications observed in SLP76-S376A T cells affect their function, we initially analyzed proliferation and activation-induced cell death of *in vitro*-activated T cells.

**Fig 5:**
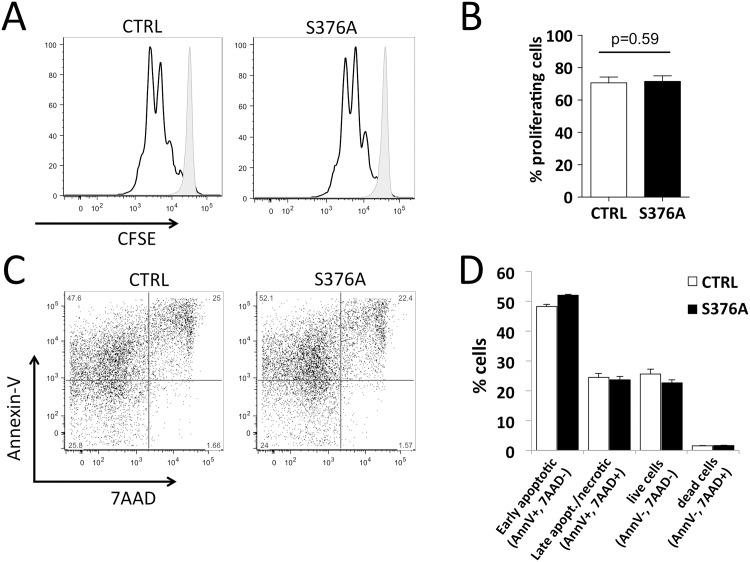
Ser376 mutation does not alter proliferation or activation-induced cell death of T cells. **A.** Purified CD4+ T cells from control (CTRL, left panel) or SLP76-S376A mice (S376A, right panel) were labeled with CFSE then stimulated for 3 days *in vitro* with anti-CD3 plus anti-CD28 antibodies as in Fig 3. Cells were then analyzed by flow cytometry to assess the dilution of CFSE of proliferating cells (empty histograms). Fluorescence of CFSE in unstimulated cells is shown as reference (filled histograms). **B**. The percentage of proliferating cells, assessed as outlined in panel A, was measured in four independent experiments, each run in three or four replicates. The histogram shows mean and SEM of pooled data from these experiments. P-value, calculated using a Mann-Whitney test, is shown above the histogram. **C.** CD4^+^ T cells stimulated as in A were stained with 7AAD and Annexin V-PE conjugate and analyzed by flow cytometry. **D.** Quantification of 7AAD and Annexin V staining of cells analyzed as described in B. Data are representative of three independent experiments. Error bars: SD.

CD4+ T cells were purified from lymph nodes, stained with CFSE and stimulated with anti-CD3 and anti-CD28 antibodies as explained above. After 3 days, CFSE dilution was measured by flow cytometry to quantify the proliferative activity. As shown in Fig 5A and 5B, CFSE dilution profiles of wild-type and SLP76-S376A T cells were comparable, with no significant difference in the percentage of proliferating cells upon stimulation. In similar experiments, cells stimulated for 3 days *in vitro* were stained with Annexin-V and 7AAD for evaluating activation-induced cell death. Staining of surface-exposed phosphatidylserine by Annexin-V together with detection of loss of membrane integrity by the DNA intercalating agent 7-AAD was used to distinguish early apoptosis from late apoptosis/necrosis. These experiments revealed that activation-induced cell death was comparable in mutant and control T cells (Fig 5C and 5D).

These results demonstrate that SLP76-S376A mutation does not affect TCR-dependent proliferation and apoptosis induced by TCR stimulation *in vitro*. Interestingly, they also reveal that, despite multiple similarities in early TCR dependent signaling, SLP76 Ser376 mutation in mice has a different outcome than HPK1 knockout, since both T cell proliferation and cell death were altered by the latter [21, 22].

### SLP76-S376A alters TCR-induced cytokine production by CD4^+^ T cells

**Fig 6:**
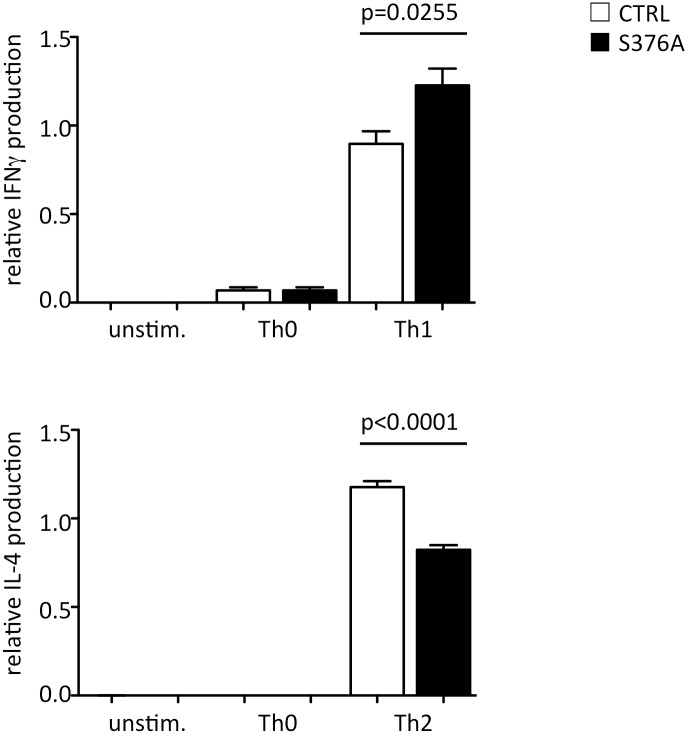
Cytokine secretion by *in vitro* differentiated and restimulated SLP76-S376A T cells. Naïve CD4+ T cells from control (empty bars) or SLP76-S376A mice (filled bars) were stimulated by anti-CD3 and anti-CD28 antibodies *in vitro* for 5 days in non-polarizing (Th0) or Th1 or Th2 polarizing conditions (see Materials and Methods). Cells were then washed and restimulated with antibodies for 24 h. Secretion of IFNγ (top panel) and IL-4 (bottom) was assessed in culture supernatants by ELISA. Data represents mean+SEM from five independent experiments, each run at least in duplicate. Statistical significance was assessed by a Mann-Withney test.

In order to further evaluate the potential consequences of SLP76-S376A mutant on T cell activation, we stimulated naïve CD4^+^ T cells under neutral conditions, or in Th1- or Th2- polarizing conditions. After five days in culture, cells were re-stimulated for 24h by anti-CD3 and anti-CD28 antibodies and specific cytokine secretion was measured by ELISA. As shown in Fig 6, cells maintained in non-polarizing conditions during primary stimulation produced low amounts of IFNγ, whereas IL4 secretion was undetectable. Conversely, cells stimulated in Th1-polarizing conditions robustly produced IFNγ. Interestingly, SLP76-S376A T cells secreted more IFNγ than control cells (Fig 6A) under these conditions. Likewise, IL-4 secretion was readily detected after restimulation of cells differentiated toward a Th2 phenotype. In this case, the amount of cytokine produced by SLP76-S376A T cells was significantly decreased relative to control T cells (Fig 6B).

Thus, these data suggest that signaling modulation by SLP76-S376A induces a shift in functional response of T cells, favoring the induction of Th1 cytokines while reducing Th2 cytokine production.

## Discussion

In this article, we present a characterization of a knock-in mouse strain expressing the mutant adaptor protein SLP76-S376A. Based on our previous work in human T cell lines and primary T cells, we expected this mutation to impair the negative feedback loop driven by HPK1-dependent phosphorylation of SLP76 at Ser376 and recruitment of 14-3-3 proteins. This, in turn, would result in increased TCR-dependent signaling and activation. Data presented herein confirm that this regulatory loop is relevant in TCR-stimulated murine T cells. Indeed, tyrosine phosphorylation of key signaling effectors, e.g. SLP76 (Tyr128) and PLCγ1, is increased in SLP76-S376A T cells, similarly to what was observed in SLP76-S376A-expressing or HPK1-silenced Jurkat-derived T cells [13]. Moreover, phosphorylation of AKT and ERK1/2 kinases is increased in CD4^+^ T cells from SLP76-S376A mice, unveiling the effects of Ser376 mutation on PI3K-dependent and MAPK pathways.

Analogous signaling modifications were previously shown to affect T cell functions to different extents in human T cell lines [13], as well as in T cells form HPK1-knockout mice [21, 22]. Similar to the latter, SLP76-S376A mutation results in hyper-activation of ERK kinases, but not JNK or p38, in mouse T cells. This result supports a model whereby HPK1 exerts at least part of its inhibitory effect on proximal TCR signals through Ser376 phosphorylation and 14-3-3 recruitment. Nonetheless, the outcome of T cell activation does not appear to be identical in these two mouse strains, since TCR-induced proliferation and activation-induced cell death susceptibility do not appear to be modified in SLP76-S376A mice, in stark contrast to HPK1 knockout T cells [21, 22]. Moreover, a differential effect on Th1 and Th2 cytokine production was observed, the former being increased and the latter decreased upon *in vitro* stimulation, whereas both cytokines were strongly inhibited by HPK1 knockout [21]. Thus, qualitative and/or quantitative differences in the impact of these mutations on downstream signaling and functional responses do exist, and SLP76-S376A mutation does not phenocopy HPK1 knockout T cells. Therefore, we conclude that HPK1-dependent negative regulation of T cell activation is predominantly driven by modifications of other signaling pathways independent of SLP76 serine phosphorylation.

The signaling pathways affected in SLP76-S376A T cells are important for transducing pre-TCR and TCR-induced signals in thymocytes and might in principle impact thymic development [3, 23, 24]. However, we were unable to identify significant alterations in numbers or phenotype of thymocytes and peripheral T cells in mutant mice. This was not completely unexpected, since thymic development appeared normal even in HPK1-deficient mice [21, 22]. Likewise, we could not detect alterations in other lymphoid or myeloid cell lineages in which SLP76 is expressed, suggesting that SLP76-S376A expression does not modify significantly developmental or homeostatic signals essential for any of these cell types. However, it cannot be ruled out that minor changes occurring in developing T or B cells have been compensated by adaptation in antigen receptor repertoires. Taken together, our data suggest that the negative regulation dependent on 14-3-3-protein interaction with SLP76 does not affect the homeostasis of hematopoietic cells.

Our previous work revealed that 14-3-3 protein dimer recruitment in SLP76-containing signaling complexes was dependent not only on binding to phosphorylated Ser376 in SLP76 but also on the interaction with a phosphorylated residue in GADS (Thr262 in human GADS, Thr254 in the mouse protein) [16]. One may argue that the lack of functional effects in SLP76-S376A T cells is due to residual recruitment of 14-3-3 to the signaling complex through GADS. However, we show now that mutation of Ser376 is sufficient to virtually abolish 14-3-3 binding to SLP76 signaling complex in mouse T cells (see Fig 3A). Why GADS phosphorylation seems to play a minor role in this system as compared to the one we described in Jurkat cells is unclear. This partial discrepancy may be due to a difference between mouse and human T cells e.g. in the relative phosphorylation levels of the two binding sites, or to an altered complex stoichiometry when overexpressing SLP76 and GADS mutants in Jurkat cells [16]. Even though a complete understanding of the contribution of GADS would require further investigation, the data presented herein indicate that Ser376 phosphorylation acts as a gatekeeper of 14-3-3 recruitment in TCR-stimulated mouse T cells [25].

Unexpectedly, wild-type and SLP76-S376A T cells displayed comparable proliferation and sensitivity to activation induced cell death. These results suggest that downregulation of signaling induced by 14-3-3 recruitment to SLP76 signaling complexes is either dispensable for modulating these T cell responses or has extremely mild effects and cannot be detected under the stimulatory setting we used. Nonetheless, we observed limited but significant alterations in cytokine production by SLP76-S376A T cells. The mechanism leading to increased IFNγ secretion and decreased IL-4 secretion by Th1 and Th2 cells, respectively, is unclear. However, our findings are compatible with models proposing a role of TCR signal strength in determining the balance between Th1 vs Th2 differentiation, predicting that stronger TCR signaling favor Th1 responses [26].

Assessing the impact of these functional differences in the regulation of immune responses will require further experiments, including challenging SLP76-S376A with specific pathogens. Modulatory effects due to Ser376 phosphorylation may become evident in particular situations, for instance during chronic antigenic stimulation or in particular inflammatory conditions (e.g. in the presence of high levels of prostaglandin E2 that activates HPK1; [22]) In these settings, this mechanism could play some role in desensitizing the TCR by sequestering SLP76 (and GADS) away from signaling complexes. Conversely, HPK1-dependent regulation of SLP76 function may be difficult to demonstrate because of its redundancy with respect to other negative-regulatory mechanisms acting on SLP76-containing signalosomes. For instance, a role for LAT ubiquitylation in downregulating T cell signaling has been recently reported [12]. Thus, if LAT inactivation occurs prior to HPK1-dependent dissociation of SLP76 from LAT, the impact of the latter mechanism would be reduced. Of note, transgenic expression of an ubiquitylation-resistant LAT mutant has limited functional differences *in vitro*, and does not appear to induce significant modifications of T cell responses *in vivo* [27]. These observations further suggest the existence of multiple redundant layers of negative regulation on the TCR signalosome.

In conclusion, our present work confirms a role for the HPK1/14-3-3/SLP76 regulatory circuit in the modulation of TCR-dependent signaling in mature T cells, and provide evidence of its effect on helper T cell responses. Nonetheless, our data highlight that, despite some overlap, Ser376 phosphorylation does not transduce all HPK1-dependent functional effects in T cells.

## Supporting Information

S1 Fig Generation of knock-in mice expressing a SLP76-S376A mutant. **A**. Strategy used to produce SLP76-S376A knock-in mice. Top panel: partial restriction map of a portion of the *Lcp2* (*Slp76*) gene surrounding exon 17. Exons are shown as filled black boxes (dark blue for exon 17) and numbered. The 5’- and 3’- probes used to verify proper homologous recombination events by Southern blot analysis are shown in red. Position of relevant *EcoRI* and *XbaI* restriction sites is also indicated. Middle panel: targeting vector used for homologous recombination. A BAC containing a region of the murine *Lcp2*, encompassing exons 15 to 20, was subcloned in a pBluescriptII KS+ vector and a T to G mutation was introduced by PCR in exon 17 to change Ser376 to Ala. At the same time, two additional silent point mutations were introduced in adjacent codons to create an *AfeI* restriction site used for screening purposes. The sequence bearing mutated exon 17 (hatched box) was then cloned in the targeting vector containing a *lox*P-tACE-CRE-PKG-gb2-*neo*^r^ cassette (see Methods). The tACE-CRE-PGK-gb2-*neo*^r^ sequence was enclosed by *lox*P sites (triangles) and directed its own excision in the male germline. TK: thymidine kinase expression cassette. (3) Structure of the targeted *Slp76-S376A* allele following homologous recombination. **B.** Southern blot analysis demonstrating appropriately recombined ES clones. ES cell DNA was digested with *EcoRI* (left panel) or *XbaI* (middle panel) and hybridized with either 5’- or 3′- single-copy probe, respectively (see A). Insertion of the CRE-*loxP* cassette was also verified by probing *XbaI* digests with a neomycin resistance gene probe (*neo* probe, right panel). **C.** Analysis of SLP76 expression and phosphorylation in lymph node T cells from wild type (CTRL) and SLP76-S376A (S376A) mice. Cells were isolated and stimulated by anti-CD3 crosslinking or calyculin A as described in legend to Fig 2. After lysis, protein extract were analyzed by gel electrophoresis and immunoblotting with the indicated antibodies. Absence of Ser376 phosphorylation in SLP76-S376A cells confirms mutation of this residue. Immunoblotting the same membrane with anti-β-tubulin was used to demonstrate comparable loading in all lanes. **D.** SLP76 protein expression in control or SLP76-S376A peripheral T cells. Lymph node T cell lysates from control (CTRL) and mutant mice (S376A) were analyzed by immunoblotting with SLP76 antibodies as in C, then reprobed with β-tubulin antibodies. SLP76 band intensity was quantified and normalized by the intensity of β-tubulin in the same sample. Relative protein amount was calculated by dividing by the average normalized SLP76 intensity in each experiment. Histogram shows mean and standard deviation of 6 independent experiments. Statistical significance was assessed by a two-tailed t-test.
(JPG)

S2 Fig Multicolor flow cytometry analysis of thymocyte sub-populations in wild-type and SLP76-S376A mice. Thymocytes isolated from wild-type (CTRL) or SLP76-S376A mice (S376A) were stained for the identification of major thymocytes subsets of αβ and γδ T cells as well as NK cell precursors and analyzed by flow cytometry. Radar plot represents percentage of total live thymocytes or percentage of parent population. Parent population is given. Mean frequencies of thymocyte subsets for CTRL and S376A mice are expressed in blue and orange respectively. TN: Triple Negative (CD3^-^CD4^-^CD8^-^); DP: Double Positive (CD4^+^CD8^+^); DP im: (immature DP (CD4^+^CD8^+^TRCβ^-^); DP sr: DP small resting (CD4^+^CD8^+^CD71^-^CD69^-^); SP: Single Positive CD4^+^ or CD8^+^); early SP (CD24^+^) or late SP (CD24^-^/lo). See also S2 Table for cell marker definitions.
(PDF)

S1 Table Antibodies used in this study for immunophenotyping of thymus and spleen. Clone numbers and providers are indicated.
(PDF)

S2 Table Marker definition of immune cell populations. The markers used to identify each population of thymic or splenic cells shown in Fig 2 and S2 Fig are indicated.
(PDF)
