# Supplementary figures and images for: Serine Phosphorylation of SLP76 Is Dispensable for T Cell Development but Modulates Helper T Cell Function
Source: PLoS One. 2017 Jan 20;12(1):e0170396. doi: 10.1371/journal.pone.0170396 (PMC5249077; doi:10.1371/journal.pone.0170396)

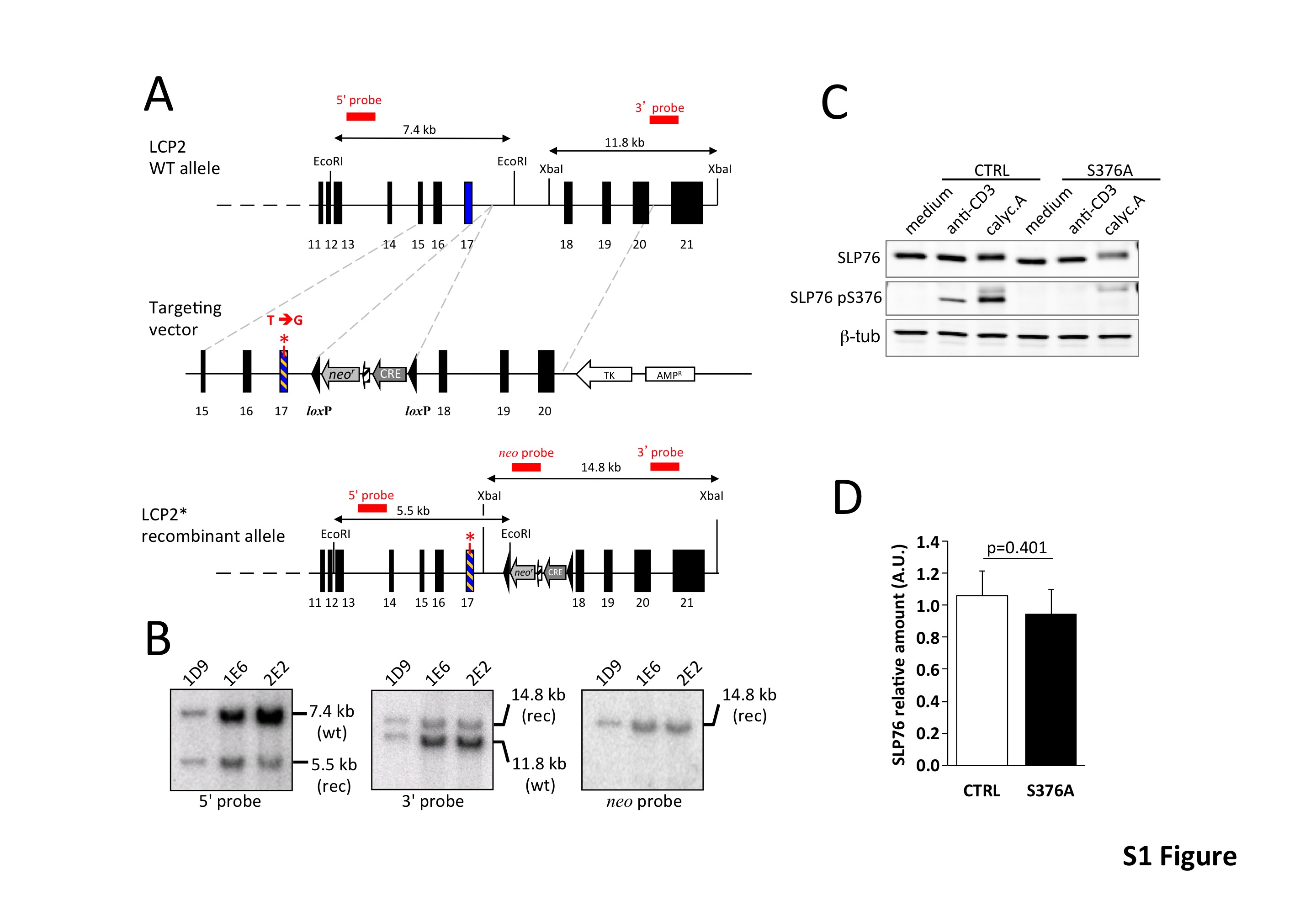

Supplement: S1 Fig — A. Strategy used to produce SLP76-S376A knock-in mice. Top panel: partial restriction map of a portion of the Lcp2 (Slp76) gene surrounding exon 17. Exons are shown as filled black boxes (dark blue for exon 17) and numbered. The 5’- and 3’- probes used to verify proper homologous recombination events by Southern blot analysis are shown in red. Position of relevant EcoRI and XbaI restriction sites is also indicated. Middle panel: targeting vector used for homologous recombination. A BAC containing a region of the murine Lcp2, encompassing exons 15 to 20, was subcloned in a pBluescriptII KS+ vector and a T to G mutation was introduced by PCR in exon 17 to change Ser376 to Ala. At the same time, two additional silent point mutations were introduced in adjacent codons to create an AfeI restriction site used for screening purposes. The sequence bearing mutated exon 17 (hatched box) was then cloned in the targeting vector containing a loxP-tACE-CRE-PKG-gb2-neor cassette (see Methods). The tACE-CRE-PGK-gb2-neor sequence was enclosed by loxP sites (triangles) and directed its own excision in the male germline. TK: thymidine kinase expression cassette. (3) Structure of the targeted Slp76-S376A allele following homologous recombination. B. Southern blot analysis demonstrating appropriately recombined ES clones. ES cell DNA was digested with EcoRI (left panel) or XbaI (middle panel) and hybridized with either 5’- or 3′- single-copy probe, respectively (see A). Insertion of the CRE-loxP cassette was also verified by probing XbaI digests with a neomycin resistance gene probe (neo probe, right panel). C. Analysis of SLP76 expression and phosphorylation in lymph node T cells from wild type (CTRL) and SLP76-S376A (S376A) mice. Cells were isolated and stimulated by anti-CD3 crosslinking or calyculin A as described in legend to Fig 2. After lysis, protein extract were analyzed by gel electrophoresis and immunoblotting with the indicated antibodies. Absence of Ser376 phosphorylation i [file pone.0170396.s001.jpg]

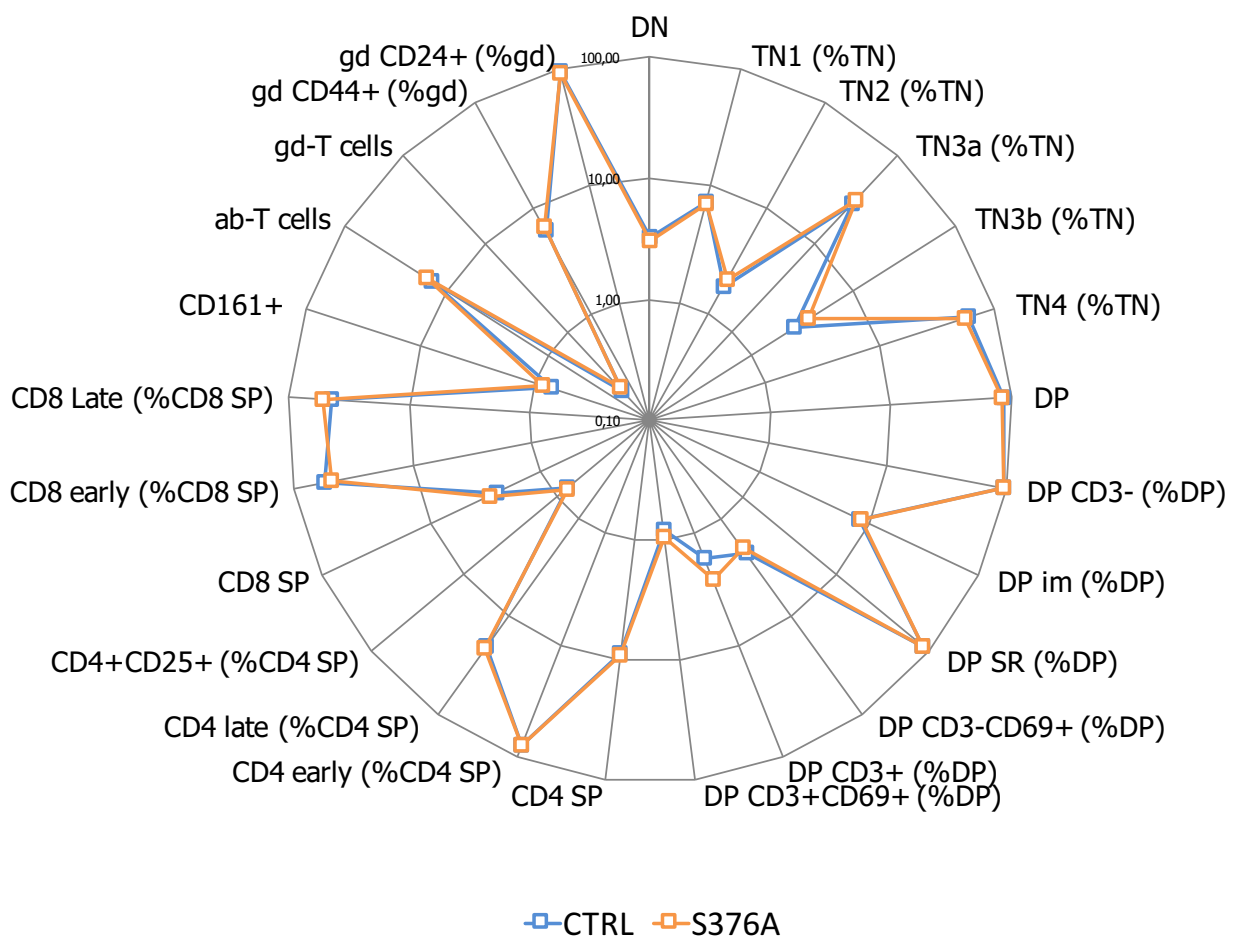

**S2 Figure**

Supplement: S2 Fig — Thymocytes isolated from wild-type (CTRL) or SLP76-S376A mice (S376A) were stained for the identification of major thymocytes subsets of αβ and γδ T cells as well as NK cell precursors and analyzed by flow cytometry. Radar plot represents percentage of total live thymocytes or percentage of parent population. Parent population is given. Mean frequencies of thymocyte subsets for CTRL and S376A mice are expressed in blue and orange respectively. TN: Triple Negative (CD3-CD4-CD8-); DP: Double Positive (CD4+CD8+); DP im: (immature DP (CD4+CD8+TRCβ-); DP sr: DP small resting (CD4+CD8+CD71-CD69-); SP: Single Positive CD4+ or CD8+); early SP (CD24+) or late SP (CD24-/lo). See also S2 Table for cell marker definitions. (PDF) [file pone.0170396.s002.pdf]
