# Supplementary material for: Serine Phosphorylation of SLP76 Is Dispensable for T Cell Development but Modulates Helper T Cell Function
Source: PLoS One. 2017 Jan 20;12(1):e0170396. doi: 10.1371/journal.pone.0170396 (PMC5249077; doi:10.1371/journal.pone.0170396)
Supplement: S1 Table — Clone numbers and providers are indicated. (PDF) [file pone.0170396.s003.pdf]

Antigen	Clone	Company
CD4	GK1.5	BD Bioscience
CD5	53-7.3	BD Bioscience
CD19	6D5	BD Bioscience
CD23	B3B4	eBioscience
CD24	M1/69	BD Bioscience
CD25	PC61	Biolegend
CD27	LG.3A10	BD Bioscience
CD38	90	eBioscience
CD43	S7	BD Bioscience
CD44	IM7	Biolegend
CD64	X54-5/7.1	Biolegend
CD69	H1.2F3	Biolegend
CD71	C2	BD Bioscience
CD93	AA4.1	Biolegend
CD95	JO2	BD Bioscience
CD103	2E7	Biolegend
CD117	2B8	BD Bioscience
CD161	PK136	BD Bioscience
CD192	475301	R&D
CD317	927	Biolegend
CD11b	M1/70	BD Bioscience
CD11c	HL3	BD Bioscience
CD16/32	2.4G2	eBioscience
CD21/35	7G6	BD Bioscience
CD3ε	145-2C11	eBioscience
CD45R	RA3-6B2	Biolegend
CD62L	MEL-14	Biolegend
CD8α	53-6.7	Biolegend
F4/80	BM8	Biolegend
IgD	11-26c.2a	Biolegend
IgM	RMM-1	Biolegend
KLRG1	2F1	Biolegend
Ly6C	HK1.4	Biolegend
Ly6G	1A8	BD Bioscience
MHCII	M5/114.15.2	Biolegend
SigF	E50-2440	BD Bioscience
TCRδ	GL3	eBioscience

S1 Table
