# Supplementary material for: Serine Phosphorylation of SLP76 Is Dispensable for T Cell Development but Modulates Helper T Cell Function
Source: PLoS One. 2017 Jan 20;12(1):e0170396. doi: 10.1371/journal.pone.0170396 (PMC5249077; doi:10.1371/journal.pone.0170396)
Supplement: S2 Table — The markers used to identify each population of thymic or splenic cells shown in Fig 2 and S2 Fig are indicated. (PDF) [file pone.0170396.s004.pdf]

Population	Organ	Marker definition
NK cells	Thymus	CD161+CD3ε-
DP Cells	Thymus	CD161-CD4+CD8α+
CD4 SP	Thymus	CD161-CD4+CD8α-
CD8 SP	Thymus	CD161-CD4-CD8α+
TN	Thymus	CD161-CD4-CD8α-CD3ε-TCRδ-
TN1	Thymus	CD161-CD4-CD8α-CD3ε-TCRδ-CD44+CD25-
TN2	Thymus	CD161-CD4-CD8α-CD3ε-TCRδ-CD44+CD25+CD24+CD117+
TN3a	Thymus	CD161-CD4-CD8α-CD3ε-TCRδ-CD44-CD25+CD24+CD71-
TN3b	Thymus	CD161-CD4-CD8α-CD3ε-TCRδ-CD44-CD25+CD24+CD71+
TN4	Thymus	CD161-CD4-CD8α-CD3ε-TCRδ-CD44-CD25-CD24+
im DP	Thymus	CD161-CD4+CD8α+CD3ε-TCRδ-CD44-CD25-CD24+CD71+
small resting DP	Thymus	CD161-CD4+CD8α+CD3ε-TCRδ-CD44-CD25-CD24+CD71-
CD3- CD69+ DP	Thymus	CD161-CD4+CD8α+CD3ε-TCRδ-CD44-CD25-CD24+CD71-
CD3+ CD69+ DP	Thymus	CD161-CD4+CD8α+CD3ε+TCRδ-CD44-CD25-CD24+CD71-
CD3+ CD4 SP	Thymus	CD161-CD4+CD8α-CD3ε+TCRδ-CD44-
Treg	Thymus	CD161-CD4+CD8α-CD3ε-TCRδ-CD44-CD25+
CD4 Early	Thymus	CD161-CD4+CD8α-CD3ε-TCRδ-CD44-CD25+CD24hi
CD4 Late	Thymus	CD161-CD4+CD8α-CD3ε-TCRδ-CD44-CD25+CD24lo
CD3+ CD8 SP	Thymus	CD161-CD4-CD8α+CD3ε+TCRδ-CD44-CD25-CD24+
CD8α Early	Thymus	CD161-CD4-CD8α+CD3ε+TCRδ-CD44-CD25-CD24+CD103-
CD8α Late	Thymus	CD161-CD4-CD8α+CD3ε+TCRδ-CD44-CD25-CD24loCD103+
γδ-T Cells	Thymus	CD161-CD4-CD8α+CD3ε+TCRδ+
Cluster A γδ+	Thymus	CD161-CD4-CD8α+CD3ε+TCRδ+CD44+CD24-
Cluster B γδ+	Thymus	CD161-CD4-CD8α+CD3ε+TCRδ+CD44-CD24+
NK cells	Spleen	CD161+CD5-
T cells	Spleen	CD161-CD5+
αβ-T	Spleen	CD161-CD5+TCRδ-
CD4+ SP	Spleen	CD161-CD5+TCRδ-CD4+
Treg (%)	Spleen	CD161-CD5+TCRδ-CD4+CD25+GITRhi
CD8 SP	Spleen	CD161-CD5+TCRδ-CD8+
γδ-T	Spleen	CD161-CD5+TCRδ+
NKT	Spleen	CD161+CD5+
B cells	Spleen	CD19+
B Mem	Spleen	CD19+CD45R+MHCII+CD38+CD23loCD21/35-IgM+IgDlo
Ag experienced	Spleen	CD19+CD45R+MHCII+CD38+CD23intCD21/35intIgM+IgD+CD71+
MZB	Spleen	CD19+CD45R+MHCII+CD38+CD23loCD21/35hiIgM+IgDlo
Plasmablastes	Spleen	CD19+CD45R+MHCII+CD38-CD95+CD71+
pre-B	Spleen	CD19+CD45RloMHCII+CD38+CD5-CD43+
Neutrophils	Spleen	CD5-CD11b+Ly6G+
Eosinophils	Spleen	CD5-Ly6G-SigF+SSChi
Monocytes	Spleen	CD5-Ly6G-SigF-SSCloCD11b+Ly6C+
Macrophages	Spleen	CD5-Ly6G-SigF-SSCloCD11b+Ly6C-MHCII+/-F4/80+CD64+CD16/32+
pDC	Spleen	CD5-Ly6G-SigF-SSCloCD11b+Ly6C-MHCII-F4/80-CD317+
cDC	Spleen	CD5-Ly6G-SigF-SSCloCD11b+Ly6C-MHCII+F4/80-CD317-CD11c+

S2 Table
